# A longitudinal study on how implicit attitudes and explicit cognitions synergistically influence physical activity intention and behavior

**DOI:** 10.1186/s40359-018-0229-0

**Published:** 2018-04-25

**Authors:** Carolin Muschalik, Iman Elfeddali, Math J. J. M. Candel, Hein de Vries

**Affiliations:** 1 Department of Health Promotion, Care and Public Health Research Institute (Caphri), Maastricht University, PO Box 616, 6200 MD Maastricht, The Netherlands; 2 GGz Breburg, Academic Department of Specialized Mental Health Care, Tilburg, The Netherlands; 3 Tilburg University, Tranzo - Scientific Center for Care and Welfare, Tilburg, The Netherlands; 4 Department of Methodology and Statistics, Care and Public Health Research Institute (Caphri), Maastricht University, Maastricht, The Netherlands

**Keywords:** Physical activity, Intention, Explicit cognitions, Implicit attitude, Interactions, Behavior change

## Abstract

**Background:**

Strategies to promote physical activity (PA) focus mainly on changing or fostering explicit cognitions and are only modestly effective. Contemporary studies suggest that, as well as explicit cognitions, implicit cognitions influence health behavior, such as PA, and that implicit processes interact with the intention to be active. Relatively little is known about whether implicit processes interact with other explicit cognitions which determine PA intention and behavior, i.e. self-efficacy. The aim of the current study was to investigate the direct effects of explicit cognitions and implicit attitudes on PA behavior as well as interactions between them regarding intention and behavior prediction.

**Methods:**

In a longitudinal study, participants (*N* = 340) completed self-report measures of explicit cognitions (perceived pros, perceived cons, social norms, social modeling, self-efficacy, intention) and activity levels, as well as a Single-Category Implicit Association Task to measure implicit attitudes towards PA at baseline (T0), and at one (T1) and 3 months thereafter (T2).

**Results:**

Hierarchical multiple regressions revealed that T0-positive implicit attitudes moderated the relationship between T0 self-efficacy and T1 PA. Similarly, T0-neutral implicit attitudes were associated with the relationship between T0 intention and T1 PA. Negative implicit attitudes strengthened the negative relationship between perceived cons and intention at baseline; neutral or positive implicit attitudes strengthened the positive relationship between self-efficacy and intention. At the follow-ups, the relationship between social modeling and intention was strengthened by negative implicit attitudes.

**Conclusion:**

This study revealed important insights into how implicit attitudes and explicit cognitions synergistically predict PA intention and behavior. As well as targeting explicit cognitions, steering a person’s implicit attitude towards a more positive one, i.e. by implicit cognitive trainings, could help to increase both PA intention and behavior.

**Electronic supplementary material:**

The online version of this article (10.1186/s40359-018-0229-0) contains supplementary material, which is available to authorized users.

## Background

Insufficient physical activity is known to cause non-communicable diseases such as hypertension, obesity, cancer, type 2 diabetes, and cardiovascular diseases [1–3]. Consequently, the need to promote physical activity (PA) has become an important public health goal [4]. Yet, the recommended level for PA – i.e. to be at least moderately physically active for 150 min per week [5] - is still not met by 31% of the world’s population [6]. To help develop more effective interventions, it is necessary to gain deeper insight into the determinants that predict PA. There are two paradigms that can be applied to explain health behaviors. The first focuses on identifying explicit beliefs of people concerning a behavior, and is inspired by a set of complementary social cognitive and ecological models which summarize multiple levels of influences on behavior [7–9]. Explicit beliefs are determinants which people are aware of, can express consciously, and are measured by self-reported questionnaires. For instance, the explicit attitude towards a behavior (e.g. ‘Being physically active is very good for my health’) or a person’s reported ability to engage in a behavior when being confronted with challenging situations, called self-efficacy (e.g. ‘I find it hard to be sufficiently physically active when I am stressed’ or ‘I find it hard to be sufficiently physically active when I dislike the activity’). The second paradigm focuses on unconscious processes which persons may not be aware of but which still influence their behavior, called implicit processes [10, 11]. Implicit attitudes are one type of implicit process. They are automatically occurring attitudes of which people are less aware and to which people do not initially have conscious access [12]. To assess implicit attitudes, computerized reaction time tasks are used, i.e. the Implicit Association Test (IAT) [13]. While several studies have applied both the explicit and the implicit paradigms, only a few focus on how to combine these approaches. The present study attempts to integrate them.

An example of the explicit paradigm is reflected by the I-Change Model [14] which has also been used to assess and change PA-related cognitions and behaviors [15–17]. According to the I-Change Model – which integrates aspects from socio-cognitive models, i.e. the Theory of Planned Behavior [9], the Trans Theoretical Model [18], Social Cognitive Theory [8] and Goalsetting Theory [19] – intention is one of the most proximal conscious determinant for behavior. Intention in turn is determined by the attitude to the behavior (comprised of perceived pros and perceived cons regarding a behavior, e.g. ‘When I am sufficiently active I have more energy’ or ‘Being sufficiently active costs me a lot of effort’), social influence (the perception of the norms and behavior of people in the social environment as well as the perceived social support, e.g. ‘Most of my friends think that I need to be sufficiently active’ or ‘Most of my friends are sufficiently active’) and self-efficacy (whether a person perceives him or herself as capable of performing a behavior when confronted with obstacles). Individuals with high levels of self-efficacy are more likely to exert effort to perform a behavior and are therefore more likely to succeed, whereas people with low levels are more likely to fail [20]. PA behavior indeed has a reliable correlation with intention, and intention in turn acts as a mediator between the explicit cognitions such as attitude, knowledge, self-efficacy, social norms and behavior and self-efficacy also has a direct effect on PA behavior [21–25]. In most of the publications on PA, this paradigm is the most dominant one and most interventions aim to increase PA levels by changing explicit cognitions. A review concludes, however, that this approach is only modestly effective [26], and the contemporary idea is that implicit cognitions need to be taken into account, in addition to explicit cognitions.

The relatively new concept of combining implicit and explicit cognitions is reflected in dual process models [10, 11, 27, 28]. According to the Reflexive-Impulsive Model (RIM) [10], an example of a dual-process model, an impulsive and a reflective system exist, both of which guide behavior. Whereas the reflective system is composed of reasoned, deliberate, and conscious motives, the impulsive system is a composition of affective responses and automatically associated behavioral tendencies. According to the RIM, the reflexive and impulsive systems can influence behavior in different ways. One way is the *double dissociation pattern* [29], according to which spontaneous behavior is predicted best by the impulsive system, and deliberate behavior by the reflexive system [30–33]. Another potential way of how the two types operate is referred to as the *additive pattern* [29], meaning that both systems explain unique variance in one behavior. This pattern has indeed been shown for purchasing healthy food [34], dental flossing [35] and also with regard to PA. Concerning the latter behavior, it has been demonstrated that automatic, less conscious processes play a unique role alongside explicit cognitions in explaining past [36, 37] and future PA behavior [38] as well as the maintenance of PA [39, 40]. From this perspective, it follows that PA is regulated by both impulsive (or implicit) and reflective (or explicit) cognitions. This conclusion was indeed reached in a recent review [41].

Although explicit and implicit constructs have been shown to play a role in determining PA, it is not clear which of the above-stated patterns can be applied to PA. Conroy and colleagues [38] showed that implicit and explicit cognitions explain unique variance in PA behavior, i.e. favoring the *additive pattern*. Berry and colleagues [42], however, challenged this approach and concluded from their study that implicit and explicit cognitions are not only directly related to PA behavior, but that implicit attitudes interact with the intention to be active. This is in line with a third way of operating, namely the *interactive pattern*, meaning that the reflective and impulsive systems interact synergistically to predict behavior [29]. Also, Cheval [43] and colleagues found that impulsive processes interacted with PA intentions. More precisely, PA intentions predicted PA behavior when the impulsive approach tendencies toward the opposite behavior of PA, namely sedentary behavior, were low or moderate. By contrast, strong impulsive approach tendencies toward sedentary behavior blocked the effect of intention on behavior. These findings suggest that the way implicit and explicit processes jointly influence PA might be more complex than so far assumed.

Although different patterns of influence have been demonstrated, we argue that the two patterns are not necessarily mutually exclusive. Implicit attitudes and explicit determinants could both have a direct effect on behavior (*additive pattern*) and also interact with each other (*interactive pattern*). Until now, the two operating models have not been tested in a single study. Furthermore, former studies, such as the one by Cheval and colleagues [43], investigated the interactive pattern only between impulsive tendencies and the explicit construct intention. We aim to take this research approach one step further and raise the question whether implicit processes might also interact with the above-mentioned explicit cognitions that predict intention (perceived pros, perceived cons, social norms, social modeling, self-efficacy) and intention itself. Just as impulsive tendencies in the study by Cheval et al. [43] either reinforced or disinhibited the relationship between intention and behavior, we assume that implicit attitudes could have a reinforcing or inhibiting effect on the relationship between explicit cognitions and intention. For instance, it is conceivable that a person who perceives many pros regarding PA has an even stronger intention to become active when he or she unconsciously evaluates the behavior as positive. If, however, the same person evaluates PA unconsciously as negative, we expect this negative implicit attitude to inhibit the effect of perceived pros on intention. The similar pattern of reasoning could be applied to the other predictors of intention. Although intention does not necessarily lead to behavior, it still accounts for 23% of the variance in PA [44] and is regarded as an important step in the adoption and maintenance of behavior and as a good predictor in the context of protective behaviors such as PA [45]. Shedding light on the joint role that implicit attitudes and explicit cognitions play in intention formation could help to further elucidate this process.

**Fig. 1:**
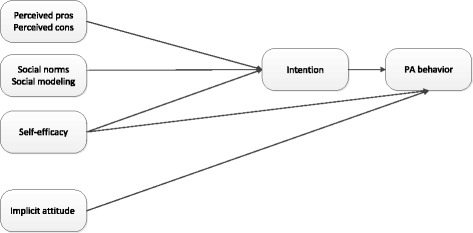
Assessing the direct effects of the explicit cognitions (perceived pros, perceived cons, social norms, social modeling, self-efficacy, intention) and the implicit attitude on PA behavior

**Fig. 2:**
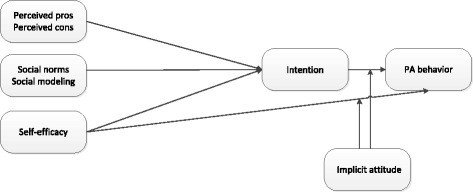
Assessing the interaction effects of implicit attitudes on the relation between self-efficacy and PA behavior and the relation between intention and PA behavior

**Fig. 3:**
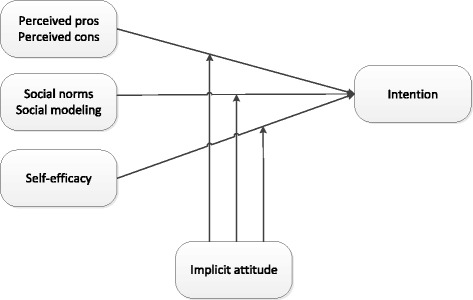
Assessing the interaction effects of implicit attitudes on the relations between perceived pros and intention, perceived cons and intention, social norms and intention, social modeling and intention, and self-efficacy and intention

The aim of the present study was three-fold. First, we investigated the direct effects of implicit attitudes and explicit cognitions on PA behavior (Fig. 1). As found in the former two studies [38, 43], we expect both implicit attitudes and explicit cognitions to predict unique variance in PA behavior (H1). Second, interactions between implicit attitudes and intention and implicit attitudes and self-efficacy were examined (Fig. 2). Just like Cheval [43] and in line with an interactive pattern of behavior prediction, we assume implicit attitudes also moderate the relationship between intention and PA and self-efficacy and PA (H2). Third, interactions between explicit cognitions and implicit attitudes were assessed (Fig. 3). We expect that the positive influence of the explicit cognitions (perceived pros, social norms, social modeling and self-efficacy) on intention is strengthened by positive implicit attitudes. The negative effect of perceived cons on intention is expected to be weakened by positive implicit attitudes but strengthened by negative implicit attitudes (H3).

## Method

### Design

A longitudinal study was conducted with a baseline measurement (T0), a follow-up after 1 month (T1) and another follow-up after 3 months (T2).

### Power analysis

With the assumption of a small effect size (f^2^ = 0.023) for a main effect or interaction effect of implicit attitude and a test power set at 0.80 with a type I error rate of α = 0.05 for two-sided testing, power analysis revealed that 330 respondents are needed. Anticipating a drop-out rate of 20%, we aimed to conduct the first session of the study with 413 participants in order to have data from at least 330 participants at the first follow-up.

### Participants and recruitment

Following approval, the study was conducted in the Behavioral and Experimental Economics Laboratory (BeeLab) of Maastricht University. Students registered in the BeeLab database were invited to participate. As most registered students were of either German or Dutch nationality, the study was conducted in these two languages. Thus, being Dutch or German was the only inclusion criterion for being invited. In total, 340 students (61% female, mean age = 21) participated in the baseline measurement. At the first follow-up, 240 students participated (71% of baseline, 64% female, mean age = 21) and a total of 128 students (38% of baseline, 69% female, mean age = 22) completed the second follow-up, 3 months after baseline.

### Procedure

Potential participants registered in the BeeLab database received an invitation email containing the following information: the study aims to gain insight into the relationships of cognitions related to PA; it consists of three waves; one measurement is comprised of 2 tasks which together take 30 min to complete; there are no expected risks associated with participation; all data will be gathered and analyzed anonymously; participants will receive 15€ in cash after the first two waves and another 7,50€ in cash after participation in the third wave. Those willing to participate could select a timeslot from two given days for each wave. One day before participating, a reminder was sent. On the day of participation, participants were welcomed in the lab, received instructions, and informed consent was obtained from all individuals included in the study. In the first part, participants performed a modified version of the Single-Category Implicit Association Test (SC-IAT) [46] to assess implicit attitudes towards PA. In the second part, participants filled in a self-report questionnaire to measure explicit cognitions and PA behavior. Explicit cognitions were assessed subsequently as a prior assessment of explicit cognitions is assumed to trigger thoughts related to PA which in turn might influence the reaction time in a following task [47]. The SC-IAT and the questionnaire were available in Dutch and in German. After completion participants were thanked and if they took part in follow-ups received their incentive at T1 and T2.

### Measurements

#### Implicit attitude assessment task

Implicit attitudes towards PA were measured with the SC-IAT. Whereas the IAT relies on the comparison of two opposite categories, e.g. men versus women, the SC-IAT does not. Regarding PA, it is difficult to define a clear opposite category as PA behavior occurs on a continuum. Moreover, the SC-IAT has proved to predict objectively-measured physical activity [38] and unintentional physical activity [38, 48]. Also, adequate internal reliability and predictive validity were demonstrated [46].

The SC-IAT consisted of two blocks, each comprising 24 practice trials and 72 test trials. In one block, “physical activity or positive” formed one category and “negative” the other category. In the other block, “physical activity or negative” was one category and “positive” the other. It is assumed that a person possesses a positive implicit attitude when he or she is quicker to categorize the displayed stimuli when “physical activity or positive” form one category than when “physical activity or negative” are one category. When this pattern is reversed, the person is assumed to hold a negative implicit attitude. The order of the two blocks was counterbalanced, meaning that the block “physical activity or positive” and “negative” had to be performed first by some participants, whereas other participants performed the block “physical activity or negative” and “positive” first. Labels for the two categories were presented on either the left or right upper part of the screen throughout the task. One by one, stimuli were presented in the centre of the screen and participants had to press *e* on their keyboard when the stimulus belonged to the category presented on the left or *i* when the stimulus belonged to the category displayed on the right. The sequence in which the stimuli were presented was randomized and words appeared an equal number of times. When an incorrect answer was given, a red X appeared on the screen until a correct answer was given.

Positive and negative words were selected from the Affective Norms for English Words (ANEW) [49] based on their valence and arousal norms. The words were translated to and from Dutch and German by German and Dutch native-speaking researchers of Maastricht University. In an informal pretest, 26 German and 22 Dutch students of Maastricht University rated the words with regard to the perceived levels of valence (1 = very negative to 9 = very positive), arousal (1 = not arousing at all to 9 = very arousing), and familiarity (1 = very unfamiliar to 9 = very familiar) in their respective mother tongue. On this basis, the following positive words were selected: love, freedom, joy, success and party (translated from German and Dutch). The selected negative words were: depression, demon, lie, infection, and poison (translated from German and Dutch). Words representing PA were carefully chosen from earlier studies in which the SC-IAT was used to assess implicit attitudes towards PA [38, 48]. These were also translated to and from German and Dutch and pretested for their representativeness for PA in both languages (1 = not representative at all, 2 = not very strongly/moderately representative, 3 = strongly representative). The seven words that were highly representative for PA were: running, biking, kickboxing, sprinting, jogging, weight-lifting, and (doing) sit-ups (translated from German and Dutch).

The SC-IAT was programmed using Inquisit by Millisecond software and the script was based on Karpinski and Steinman [46]. The implicit attitude was formed by d-scores, calculated automatically using Inquisit software by subtracting the average response time for the test block with the categories *physical activity or positive*/*negative* from the average response time of the test block with the categories *physical activity or negative*/*positive.* This score was then divided by the standard deviation of all test trials. This procedure is based on the improved scoring algorithm as described by Greenwald and colleagues [50]. D-scores can range from − 2 to + 2 with negative values representing a negative implicit attitude and positive values representing a positive implicit attitude. The higher the d-score the more positive an implicit attitude. Reliability test of the SC-IAT was calculated based on the procedure as described in Karpinski and Steinman [46] and revealed an acceptable value of *r* = .83.

#### Self-report assessment

All explicit cognitions referred to adequate physical activity. Adequate PA for adults was defined as being moderately physically active five times a week for at least 30 min. Moderately active is described as, for instance, brisk walking with an increase in heart rate [51]. This definition was presented to the participants and could be re-read at any time while answering the questionnaire. The questions to measure explicit cognitions were based on the I-Change model [14]. For the full questionnaire, see Additional file 1.

Explicit attitude was assessed using 20 items that were rated on a 5-point Likert Scale. Ten items assessed the perceived cons of adequate PA (Cronbach’s α = .83) and 10 items assessed the perceived cons of adequate PA (Cronbach’s α = .77). One example item for pros is “When I am adequately active it is” with answer options ranging from (1) “very good for my health” to (5) “not good for my health”. Items were reversed so that higher values represent the perception of more pros. An example for cons is “When I am adequately active it is” with answer options from (1) “too time-consuming” to (5) “not time-consuming”. Items were reversed, so that lower scores represent the perception of fewer cons. One scale score for perceived pros and one for perceived cons were created for the analyses.

Social norms and social modeling were assessed by four questions. Answers were given on a 5-point Likert scale and assessed the norms about adequate physical activity of family members, partners, and friends (Cronbach’s α = .74) and their PA behavior (Cronbach’s α = .48). An item representing norms was “Most of my friends” (1) “certainly think that I need to be adequately active” to (5) “certainly do not think that I should be adequately active”. An additional answer option: “I don’t have any friends/Not applicable” was given as a sixth option. A modeling item was “Most of my friends are adequately physically active” with answer options from (1) “totally agree” to (5) “totally disagree”. The additional answer option “I don’t have any friends/Not applicable” was also available. These answers were not included in the analyses. Norms and modeling items were reversed with higher scores representing stronger norms or modeling. The mean scale scores for norms and modeling were included in the analyses.

Self-efficacy was measured by nine items, also on a 5-point Likert scale (Cronbach’s α = .74). These items enquired about the extent to which respondents thought they would be able to be adequately physically active in different situations. For instance “I find it difficult/easy to be adequately physically active when I am tired” with answer options from (1) “very difficult” to (5) “very easy”. Questions were based on those used in former studies about PA [15, 52, 53]. Higher scores indicate higher self-efficacy. The mean scale score was included in the analyses.

Intention was measured by three items on a 5-point Likert scale (Cronbach’s α = .87). The first item assessed whether respondents intended to become adequately physically active within the next 3 months, ranging from (1) “yes, absolutely” to (5) “no, not at all”. The second item asked whether respondents were motivated to become adequately physically active within the next 3 months with answer options ranging from (1) “totally agree” to (5) “totally disagree”. The third item measured how high the chances were of becoming adequately physically active within the next 3 months. Answer options ranged from (1) “very little” to (5) “very high”. The first two items were reversed, so that higher scores represent a stronger intention. The mean score of all three items was included as scale score for intention in the analyses.

Physical activity levels were measured by the Short Questionnaire to Assess Health-enhancing physical activity (SQUASH). This has been proven to be a reliable and valid tool for assessing PA levels among Dutch adults [54, 55] and has been applied in former studies [15–17, 53]. Completing the SQUASH takes around 5 min; it assesses different domains of PA, namely commuting activities, activities at work, household activities, and leisure time activities. For each activity, frequency (days per week), duration (minutes per day) and intensity (light/moderate/intense expressed in metabolic equivalent of task, MET) were measured. MET values for sport activities were derived from Ainsworth and colleagues [56]. Based on the procedure of Wendel-Vos and colleagues [54], the total minutes of an activity were calculated by multiplying frequency by duration. These were then multiplied by the intensity in order to obtain an activity score for each activity. A total activity score was calculated by summing all activity scores. The higher the score, the more physically active a person is.

Additionally, participants gave information about their age, gender, use of drugs, alcohol or medications that could influence their reaction time, and whether they were able to be physically active in the recent past.

### Analyses

Differences between the German and Dutch version of the tests were tested in advance. No significant differences were found. Descriptive analyses were conducted to describe the sample. To assess whether study variables changed significantly over time, linear mixed models were used. Logistic regression analysis was used to evaluate whether dropout was predicted by age, gender, perceived pros, perceived cons, social norms, social modeling, self-efficacy. All analyses were done with SPSS version 23.

For the first hypothesis, two hierarchical multiple regressions were performed: one with PA behavior after 1 month, and a second with PA behavior after 3 months as dependent variable. Baseline variables were included as predictors in three steps. In step 1 we entered age and gender, in step 2 perceived pros, perceived cons, social norms, social modeling, self-efficacy and intention, and in step 3 implicit attitudes as predictor. For hypothesis 2, there was a fourth step, entering all interaction terms between implicit attitude and the explicit cognitions. If there were significant interaction terms, follow-up stratified analyses were conducted [57]. In this case, implicit attitude was categorized into positive, neutral, and negative based on the tertiles of its score distribution. Implicit attitude scores ≤ − .053 were categorized as negative, implicit attitude scores > − .053 and ≤ .285 were considered neutral, and scores > .285 as positive. To test whether the interactions found added significantly to the prediction of PA after 1 month or after 3 months, another hierarchical regression analysis was performed, only adding the significant interaction terms. To test hypothesis 3, hierarchical multiple regressions, similar to those carried out for question 2, were performed, but this time with intention at baseline, after 1 month and after 3 months as dependent variable. In step 1, we again entered age and gender; in step 2, perceived pros, perceived cons, social norms, social modeling, self-efficacy and implicit attitudes; and in step 3, all interaction terms between implicit attitude and the explicit cognitions. All predictors were mean-centered before entering into the models. Cases with missing values were not included in the analyses.

## Results

### Descriptives

In total, 372 students participated in the baseline measurement. Answers of 32 participants were excluded as their reaction times could not be linked to their questionnaire answers. The remaining sample was *N* = 340 (61% female, mean age = 21). Table 1 shows the characteristics of the sample and the differences over time regarding study variables. At follow-up one and two, more men dropped out than women (T1: OR = 0.55, 95% CI = 0.04–1.0, *p* = .02; T2: OR = 0.51, 95% CI = 0.02–1.0, *p* = .01). No other variables predicted dropout.

**Table 1:** Characteristics of the study sample and differences between study variables over time

	T0 (*N* = 340)	T1 (*n* = 240)	T2 (*n* = 128)	F value	df	*P* value
Sex (female), n (%)	212 (61.1)	165 (63.5)	101 (70.1)			
Age in years	21 (2.11)	21 (2.14)	21 (2.19)			
Perceived pros	4.23 (.47)	4.29 (.46)	4.30 (.47)	1.91	737	.15
Perceived cons	2.00 (.50)	2.01 (.53)	2.01 (.51)	.11	737	.89
Social norms	3.89 (.74)	3.90 (.74)	4.05 (.66)	3.06	737	.05
Social modeling	3.45 (.65)	3.43 (.71)	3.46 (.73)	.10	737	.90
Self-efficacy	2.60 (.62)	2.56 (.61)	2.59 (.65)	.53	737	.59
Implicit attitude	.116 (.331)	.130 (.338)	.141 (.325)	.63	737	.53
Intention	4.43 (.67)	4.38 (.70)	4.42 (.64)	.78	737	.46
Physical activity	4959.03 (3187.16)	5401.21 (2980.59)	5593.24 (2888.56)	3.32	737	.04

### Hypothesis 1

#### The contribution of implicit attitudes to the variance in PA behavior

Implicit attitudes did not add directly to the prediction of PA behavior after 1 month of follow-up (F_change_ (1, 230) = .04, *p* = .84), nor after 3 months’ follow-up (F_change_ (1, 118) = 1.48, *p* = .23). After 1 month, intention (*t* = 1.98, *p* = .05) and self-efficacy (*t* = 2.92, *p* = .04) explained 13% of the variance in PA behavior, and after 3 months, self-efficacy (*t* = 2.44, *p* = .02) explained 16% of the variance in PA behavior.

### Hypothesis 2

#### Moderating effects of implicit attitudes on the relationship between explicit cognitions and PA behavior

After 1 month of follow-up, the effect of self-efficacy on PA behavior was marginally but not significantly moderated by implicit attitudes (*p* = .06). The positive relationship between self-efficacy and PA was significantly strengthened when people had a positive implicit attitude (β = .411) compared to when the implicit attitude was negative (β = −.040; *p* = .02). The interaction did not add significantly to the prediction of PA at T1 (F_change_ (1, 229) = 2.69, *p* =.10). After three months, implicit attitudes moderated, although only marginally significantly, the relationship between intention and PA (*p* = .08). The relationship was stronger when people held a neutral implicit attitude (β = .376) compared to when they held a positive implicit attitude (β = −.296; *p* = .03) towards PA. The interaction did not add significantly to the prediction of PA at T2 (F_change_ (1, 117) = 1.83, *p* =.18). Table 2 shows the results for each of the four steps of the hierarchical regression.

### Hypothesis 3

#### Moderating effects of implicit attitudes on the relationship between explicit cognitions and PA intention

Interaction effects were found at baseline between perceived cons and implicit attitudes (*p* = .07) as well as between self-efficacy and implicit attitudes (*p* = .04). Table 3 presents the results for each of the four steps of the hierarchical regression.

**Table 2:** Coefficients of the hierarchical multiple regression analysis with PA at T1 and T2 as dependent variable. Interactions with implicit attitudes are added at step 4

Block	Independent variable	PA at T1					PA at T2				
		B	SE	β	p	R^2^	B	SE	β	p	R^2^
1	Gender	157.43	401.10	0.03	0.70	.01	402.48	549.70	0.07	0.47	.01
	Age	159.04	89.97	0.11	0.08		126.51	117.45	0.10	0.28	
2	Gender	410.09	395.25	0.07	0.30	.13	752.31	550.60	0.12	0.17	.16
	Age	184.69	88.50	0.13	0.04		168.27	118.98	0.13	0.16	
	Perceived pros	186.54	443.57	0.03	0.67		273.97	569.21	0.05	0.63	
	Perceived cons	− 162.19	445.26	−0.03	0.72		− 282.93	562.87	− 0.05	0.62	
	Social norms	93.69	281.24	0.02	0.74		−253.44	411.51	−0.06	0.54	
	Social modeling	145.73	307.02	0.03	0.64		−327.40	416.45	−0.08	0.43	
	Self-efficacy	1018.24	348.51	0.21	0.04		1049.31	430.11	0.24	0.02	
	Intention	693.96	350.41	0.15	0.05		738.01	461.74	0.17	0.11	
3	Gender	392.83	405.72	0.06	0.33	.13	835.17	553.71	0.14	0.13	.17
	Age	185.71	88.83	0.13	0.04		149.69	119.72	0.11	0.21	
	Perceived pros	178.04	446.60	0.03	0.69		420.83	580.76	0.07	0.47	
	Perceived cons	−165.12	446.44	−0.03	0.71		− 307.65	562.11	−0.06	0.59	
	Social norms	88.48	283.07	0.02	0.75		−305.76	412.93	−0.07	0.46	
	Social modeling	156.39	312.43	0.03	0.62		−327.30	415.61	−0.08	0.43	
	Self-efficacy	1020.83	349.48	0.21	0.04		976.69	433.38	0.22	0.03	
	Intention	693.04	351.18	0.15	0.05		715.65	461.18	0.17	0.12	
	Implicit attitude	−117.58	599.03	−0.01	0.84		999.95	822.30	0.11	0.23	
4	Gender	333.74	412.23	0.05	0.42	.15	1024.46	569.58	0.17	0.07	.21
	Age	201.59	90.99	0.14	0.03		154.76	125.83	0.12	0.22	
	Perceived pros	106.07	457.43	0.02	0.82		712.90	608.82	0.12	0.24	
	Perceived cons	− 202.10	460.29	−0.03	0.66		− 339.29	594.66	−0.06	0.57	
	Social norms	111.38	287.22	0.03	0.70		− 379.21	439.25	−0.08	0.39	
	Social modeling	133.70	318.88	0.03	0.68		−435.61	430.50	−0.10	0.31	
	Self-efficacy	1000.94	352.48	0.21	0.05		943.53	439.23	0.22	0.03	
	Intention	680.71	358.98	0.15	0.06		482.54	476.19	0.11	0.31	
	Implicit attitude	31.84	610.01	0.03	0.96		1343.57	850.57	0.14	0.12	
	Perceived pros X Implicit attitude	608.53	1706.51	0.03	0.72		3803.21	2516.52	0.17	0.13	
	Perceived cons X Implicit attitude	957.04	1398.11	0.05	0.49		− 1034.64	2097.64	−0.06	0.62	
	Social norms X Implicit attitude	− 553.86	928.58	−0.04	0.55		141.89	1589.57	0.01	0.93	
	Social modeling X Implicit attitude	− 226.18	1100.33	−0.01	0.84		− 1279.73	1615.44	−0.08	0.43	
	Self-efficacy X Implicit attitude	2155.73	1157.93	0.14	0.06		− 536.38	1478.98	−0.04	0.72	
	Intention X Implicit attitude	− 729.76	1099.42	−0.06	0.51		− 2958.15	1687.82	−0.21	0.08	

The negative relationship between perceived cons and intention was significantly strengthened when people held a negative implicit attitude (β = −.368) compared to when the implicit attitude was positive (β = −.085; *p* = .03). The positive relationship between self-efficacy and intention was significantly strengthened when people held a neutral (β = .232) or a positive implicit attitude (β = .326) compared to when the implicit attitude was negative (β = −.002; *p* = .05, *p* = .01). Along with perceived pros and social modeling, the significant interactions added, although only marginally, significantly to the prediction of intention at baseline (F_change_ (2, 329) = 2.63, *p* = .07), and explained 42% of the variance in the intention to become physically active, i.e. 2% more than without the interactions.

After 1 month’ follow-up an interaction effect between implicit attitudes and social modeling was found (*p* = .02). The effect was significantly stronger when people held a negative implicit attitude (β = .359) compared to when the implicit attitude was positive (β = .050, *p* = .06). Along with perceived pros, perceived cons and self-efficacy, the interaction added significantly to the prediction of intention after 1 month, (F_change_ (1, 231) = 5.48, p = .02) and explained 32% of the variance in the intention, i.e. 1% more.

After 3 months, implicit attitudes moderated the relationship of social modeling to intention (*p* = .03). The relationship was, although only marginally significant, stronger when people held a negative (β = .378) compared to a positive implicit attitude (β = −.073; *p* = .08) to PA. Along with perceived pros and perceived cons, the interaction between social modeling and implicit attitude significantly added to the prediction of intention after 3 months (F_change_ (1, 118) = 5.08, p = .03) and explained 39%, i.e. 3% more, of the variance in the intention.

**Table 3:** Coefficients of the hierarchical multiple regression analysis with intention at T0, T1, and T2 as dependent variable. Interactions with implicit attitudes are added at step 4

Block	Independent variable	Intention at T0					Intention at T1					Intention at T2				
		B	SE	β	p	R^2^	B	SE	β	p	R^2^	B	SE	β	p	R^2^
1	Gender	−0.12	0.07	−0.09	0.10	.01	−0.09	0.09	−0.06	0.37	.01	−0.03	0.12	−0.02	0.78	.01
	Age	−0.01	0.02	−0.02	0.73		−0.01	0.02	−0.01	0.92		0.03	0.03	0.11	0.22	
2	Gender	0.00	0.06	0.00	0.98	.40	0.01	0.08	0.01	0.93	.31	−0.02	0.11	−0.02	0.85	.36
	Age	−0.02	0.01	−0.05	0.25		−0.01	0.02	−0.03	0.56		0.00	0.02	−0.01	0.95	
	Perceived pros	0.44	0.06	0.31	< 0.001		0.33	0.09	0.22	< 0.001		0.31	0.11	0.23	0.03	
	Perceived cons	−0.36	0.07	−0.27	< 0.001		−.33	0.09	−0.24	< 0.001		−0.48	0.11	−0.40	< 0.001	
	Social norms	0.01	0.04	0.01	0.74		0.02	0.06	0.02	0.80		−0.10	0.08	−0.10	0.23	
	Social modeling	0.19	0.05	0.18	< 0.001		0.17	0.06	0.16	0.01		0.24	0.08	0.25	0.03	
	Self-efficacy	0.20	0.05	0.18	< 0.001		0.23	0.07	0.20	0.01		0.02	0.08	0.02	0.85	
3	Gender	0.00	0.06	0.00	0.99	.40	0.02	0.08	0.01	0.83	.31	−0.01	0.11	−0.01	0.94	.36
	Age	−0.02	0.01	−0.05	0.25		−0.01	0.02	−0.04	0.54		0.00	0.02	−0.01	0.86	
	Perceived pros	0.44	0.06	0.31	< 0.001		0.34	0.09	0.23	< 0.001		0.33	0.11	0.25	0.02	
	Perceived cons	−0.37	0.07	−0.27	< 0.001		−0.33	0.09	−0.24	< 0.001		−0.48	0.10	−0.40	< 0.001	
	Social norms	0.01	0.04	0.01	0.75		0.02	0.06	0.02	0.76		−0.11	0.08	−0.10	0.20	
	Social modeling	0.19	0.05	0.18	< 0.001		0.16	0.06	0.15	0.01		0.24	0.08	0.25	0.003	
	Self-efficacy	0.20	0.05	0.18	< 0.001		0.23	0.07	0.20	0.001		0.01	0.09	0.01	0.96	
	Implicit attitude	−0.02	0.09	−0.01	0.85		0.08	0.13	0.04	0.53		0.15	0.16	0.07	0.36	
4	Gender	−0.01	0.06	−0.01	0.89	.42	−0.01	0.09	−0.01	0.94	.33	−0.02	0.11	−0.01	0.87	.40
	Age	−0.01	0.01	−0.04	0.34		−0.01	0.02	−0.04	0.54		0.00	0.02	−0.02	0.85	
	Perceived pros	0.44	0.06	0.31	< 0.001		0.35	0.09	0.24	< 0.001		0.35	0.11	0.26	0.002	
	Perceived cons	−0.36	0.07	−0.27	< 0.001		−0.34	0.09	−0.25	< 0.001		−0.46	0.11	−0.39	< 0.001	
	Social norms	0.01	0.04	0.01	0.80		0.02	0.06	0.02	0.79		−0.12	0.09	−0.12	0.15	
	Social modeling	0.19	0.05	0.19	< 0.001		0.14	0.07	0.14	0.03		0.23	0.08	0.24	0.01	
	Self-efficacy	0.19	0.05	0.17	< 0.001		0.22	0.07	0.19	0.003		−0.02	0.09	−0.02	0.83	
	Implicit attitude	−0.01	0.09	0.00	0.93		0.08	0.13	0.04	0.53		0.15	0.16	0.07	0.35	
	Perceived pros X Implicit attitude	−0.18	0.20	−0.04	0.37		0.01	0.31	0.01	0.97		0.48	0.45	0.09	0.30	
	Perceived cons X Implicit attitude	0.40	0.22	0.09	0.07		0.23	0.29	0.05	0.43		0.23	0.40	0.06	0.58	
	Social norms X Implicit attitude	0.05	0.13	0.02	0.69		0.22	0.19	0.07	0.25		0.24	0.31	0.07	0.45	
	Social modeling X Implicit attitude	0.05	0.15	0.02	0.72		−0.51	0.22	−0.14	0.02		−0.68	0.31	−0.19	0.03	
	Self-efficacy X Implicit attitude	0.34	0.16	0.10	0.04		0.25	0.23	0.07	0.28		0.05	0.28	0.02	0.86	

## Discussion

The present study aimed to shed light on the question how implicit attitudes influence PA intention and behavior together with well-known explicit predictors of PA. Direct effects of these variables as well as interactions between them were examined. Results showed that implicit attitudes did not have a direct effect on PA behavior albeit via other explicit cognitions. The fact that implicit attitudes did not have a direct effect on PA behavior at any measuring point is in contrast to our hypothesis as well as to earlier results of Conroy and colleagues [38] and Cheval and colleagues [43]. Both authors found that, after controlling for explicit motivational predictors, implicit processes significantly contributed to PA prediction and hence support for the *additive pattern*. Whereas above authors assessed PA behavior using pedometers, we assessed PA levels by means of a self-report questionnaire, which, despite its shown validity [54], is less accurate than direct measurements [58, 59]; this could be a reason for the non-significant findings. Follow-up studies using accelerometers may be needed to obtain further insight into whether or not implicit processes influence actual PA behavior directly.

Although we did not find any direct effects, moderating effects were demonstrated: i.e. positive implicit attitudes strengthened the positive relationship between self-efficacy and PA behavior at the first follow-up. Negative implicit attitudes were found to weaken this relationship. In addition, and similar to Cheval et al. [43], we found that neutral but not positive implicit attitudes strengthened the positive relationship between intention and PA at the second follow-up. It seems surprising that positive implicit attitudes did not strengthen the relationship between intention and PA, but this could be explained by a ceiling effect as the intention of participants to be active was already very strong. Nonetheless, the findings support the idea of an interactive pattern of influencing PA behavior which is also in line with the findings of Cheval and colleagues [43]. If the intention to be active is already strong, positive implicit attitudes do not seem to support the effect on behavior, whereas neutral implicit attitudes do. In order to strengthen the likelihood that intention translates into behavior, our results suggest that one should at least aim to diminish a negative implicit attitude and create a neutral implicit attitude.

Moreover, we found implicit attitudes moderated the relationship between several explicit cognitions and intention. Firstly, implicit attitudes moderated the relationship between perceived cons and intention as well as between self-efficacy and intention at baseline. In line with our hypothesis, negative implicit attitudes strengthened and positive implicit attitudes weakened the negative relationship between perceived cons and intention. It seems that for those participants who reported exercise not to be beneficial or pleasant (as measured by the explicitly perceived cons), the positive implicit associations with PA acted as a buffer between perceived cons and intention. Moreover, the positive relationship between self-efficacy and intention was strengthened by neutral and positive implicit attitudes. Regarding self-efficacy, it seems conceivable that the effect of intention on PA behavior is stronger when a person does not only perceive him or herself as being capable of performing the behavior, but also has a positive, or at least a neutral, unconscious attitude towards the behavior. Thus, when intending to increase PA intention, positive implicit attitudes appear to be more beneficial. The interactions were not significant at one and 3 months’ follow-up, which could either be due to the weaker power of the sample, or to the assumption that implicit attitudes only have a short-term influence on the effect of perceived cons and intention and self-efficacy and intention.

Secondly, at one and 3 months’ follow-up, implicit attitudes moderated the relationship between social modeling and intention. The impact of other people’s behavior on the intention to become physically active was significantly greater when the implicit attitude was negative compared to when it was positive. One explanation for this finding could be derived from Festinger’s cognitive dissonance theory [60], according to which, individuals seek consistency among their cognitions. When an inconsistency between attitudes or behaviors occurs, the individual is motivated to resolve it as it is accompanied by negative feelings [61]. Feeling implicitly negative about being physically active while at the same time perceiving important people in one’s environment as being physically active, might create dissonance. In order to resolve this, individuals might reduce the importance of the implicit attitude and follow the behavior of others. In this case, the explicitly perceived modeling behavior might override the implicitly perceived negative implicit association. In the present study, the negative implicit attitude had a positive effect on the relationship between social modeling and intention. However, when there is no dissonance, i.e. when a person holds a negative implicit attitude and is surrounded by people who are not sufficiently active, negative implicit attitudes might strengthen the negative relationship between social modeling and intention, as was also the case for the relationship between perceived cons and intention. As interventions may not be able to change or control behavior or the perception of peer or parent behavior, they might rather attempt to reduce the impact of these perceptions on intention by creating a positive implicit attitude. Training or changing implicit associations has been applied to reduce social anxiety [62], alcohol consumption [63], to increase implicit self-esteem [64, 65] and only recently to increase PA levels [66, 67]. While Berry and colleagues and Markland and colleagues demonstrated short-term changes in implicit attitudes via exercise imagery or the provision of (counter attitudinal) information, computerized tasks have not yet been used in this context, but might offer a fruitful alternative. More research is, therefore, needed to understand how stable and changeable implicit attitudes actually are, especially over time. Moreover, in order to understand conditions under which dissonant and congruent implicit and explicit attitudes are beneficial or detrimental for PA behavior, further research is required.

When interpreting our findings, the following possible limitations need to be taken into account. First, the study sample was quite homogenous as far as age, education, and socio-economic status were concerned and had, on average, a very positive explicit attitude and a strong intention to be physically activity, which is not representative of the general public [68]. Second, for practical reasons, PA levels were measured by self-report. It is not clear to what extent participants were explicitly aware of activities which occurred spontaneously and excluded planned, structured exercise (e.g. using the stairs), and whether they were able to report them. Despite the satisfactory validity of the SQUASH [54], supplementing it with more objective measures, such as accelerometers or pedometers, could provide a more adequate report about activity levels as suggested by other studies [58, 59]. Third, we had a high drop-out rate at our follow-up measures (29% at T1 and 62% at T2) which could be due to an absence of commitment to participate in all three measures, a panel fatigue [69] or simply due to time constraints of the student sample as the last measure was conducted shortly before the exam period. As a consequence, our sample suffered from low power after one and 3 months which makes the interpretation of (non-)findings challenging. Fourth, stratified analyses for people with a negative, neutral, or positive implicit attitude were conducted using small sub-samples; these are also likely to have suffered from low power. Fifth, although neutral or positive implicit attitudes might help to increase PA intention, the intention behavior gap still remains. Research aimed at reducing this gap should be further stimulated.

## Conclusion

Summarizing, one can conclude that the present findings challenge the dual-process approach which, until now, only assumed a direct influence of implicit attitudes on behavior, not via other explicit constructs. Although different modes of influence were suggested by Perugini [29], including an interactive pattern of influence between implicit and explicit attitudes, a thorough examination or integration of further determinants, such as intention, has not yet been carried out. Both approaches have in fact developed in isolation. We argue that this division needs to be reconsidered as our findings and those of Cheval and colleagues [43] demonstrate that unconscious processes are indeed associated with more conscious processes. A unique contribution of the present research is the examination of interactions between implicit attitudes, which are part of dual-process models, and explicit cognitions, which are summarized in socio-cognitive models. Potential improvements for interventions are thus provided. Future research needs to build on these findings by testing whether interventions which target both implicit attitudes and explicit cognitions result in greater activity intention and actual behavior change. Another avenue for future work, especially for the area of model testing and model improvement, is to investigate whether the relationships found in the present study are rather unique to PA or valid across diverse health-related behaviors. Shedding light on these issues may not only aid the development of even more successful interventions to promote physical activity, but also the aspiration to improve global health.

## Additional file

Additional file 1: English translation of the questionnaire which was used to assess the explicit cognitions perceived pros, perceived cons, social norms, social modeling, self-efficacy, and intention regarding sufficient physical activity. (DOCX 26 kb)

## Abbreviations

BeeLab: Behavioral and experimental economics laboratory
IAT: Implicit Association Test
MET: Metabolic equivalent of task
PA: Physical activity
RIM: Reflexive-Impulsive Model
SC-IAT: Single-Category Implicit Association Test
SQUASH: Short questionnaire to assess health-enhancing physical activity
T0: Baseline measure
T1: Follow-up after one month
T2: Follow-up after three months
