# Supplementary material for: A longitudinal study on how implicit attitudes and explicit cognitions synergistically influence physical activity intention and behavior
Source: BMC Psychol. 2018 Apr 25;6:18. doi: 10.1186/s40359-018-0229-0 (PMC5921561; doi:10.1186/s40359-018-0229-0)
Supplement: Supplementary file 1 — English translation of the questionnaire which was used to assess the explicit cognitions perceived pros, perceived cons, social norms, social modeling, self-efficacy, and intention regarding sufficient physical activity. (DOCX 26 kb) [file 40359_2018_229_MOESM1_ESM.docx]

| Perceived pros: items 1-10 | |
| --- | --- |
| 1. When I am adequately active it is: | (1) very pleasant |
|  | (2) pleasant |
|  | (3) somewhat pleasant |
|  | (4) barely pleasant |
|  | (5) Not pleasant |
| 2. When I am adequately active it is: | (1) very good for my health |
|  | (2) good for my health |
|  | (3) somewhat good for my health |
|  | (4) barely good for my health |
|  | (5) not good for my health |
| 3. When I am adequately active, I feel: | (1) much better |
|  | (2) better |
|  | (3) somewhat better |
|  | (4) barely better |
|  | (5) Not better |
| 4. When I am adequately active, I have: | (1) a much better [aerobic](https://www.dict.cc/englisch-deutsch/aerobic.html) [endurance](https://www.dict.cc/englisch-deutsch/endurance.html) |
|  | (2) a better [aerobic](https://www.dict.cc/englisch-deutsch/aerobic.html) [endurance](https://www.dict.cc/englisch-deutsch/endurance.html) |
|  | (3) a slightly better [aerobic](https://www.dict.cc/englisch-deutsch/aerobic.html) [endurance](https://www.dict.cc/englisch-deutsch/endurance.html) |
|  | (4) barely a better [aerobic](https://www.dict.cc/englisch-deutsch/aerobic.html) [endurance](https://www.dict.cc/englisch-deutsch/endurance.html) |
|  | (5) no better [aerobic](https://www.dict.cc/englisch-deutsch/aerobic.html) [endurance](https://www.dict.cc/englisch-deutsch/endurance.html) |
| 5. When I am adequately active, I have: | (1) much more energy |
|  | (2) more energy |
|  | (3) somewhat more energy |
|  | (4) barely more energy |
|  | (5) not more energy |
| 6. When I am adequately active, it is: | (1) very good for my mental health |
|  | (2) good for my mental health |
|  | (3) slightly good for my mental health |
|  | (4) barely good for my mental health |
|  | (5) not good for my mental health |
| 7. When I am adequately active, I find it: | (1) very enjoyable |
|  | (2) enjoyable |
|  | (3) slightly enjoyable |
|  | (4) barely enjoyable |
|  | (5) not enjoyable |
| 8. When I am adequately active, it: | (1) strongly improves my ability to think |
|  | (2) improves my ability to think |
|  | (3) slightly improves my ability to think |
|  | (4) barely improves my ability to think |
|  | (5) does not improve my ability to think |
| 9. When I am adequately active, I feel: | (1) much more attractive |
|  | (2) more attractive |
|  | (3) slightly more attractive |
|  | (4) barely more attractive |
|  | (5) not more attractive |
| 10. When I am adequately active, I can maintain my weight: | (1) much better |
|  | (2) better |
|  | (3) slightly better |
|  | (4) barely better |
|  | (5) not better |

| Perceived cons: items 1-10 | |
| --- | --- |
| 1. When I am adequately active it costs: | (1) too much time |
|  | (2) much time |
|  | (3) slightly too much time |
|  | (4) barely too much time |
|  | (5) Not too much time |
| 2. When I am adequately active, I find it: | (1) very unpleasant |
|  | (2) unpleasant |
|  | (3) slightly unpleasant |
|  | (4) barely unpleasant |
|  | (5) not unpleasant |
| 3. When I am adequately active, I get: | (1) very strong muscle pain |
|  | (2) strong muscle pain |
|  | (3) slight muscle pain |
|  | (4) little muscle pain |
|  | (5) No muscle pain |
| 4. When I am adequately active, I find it: | (1) very uncomfortable |
|  | (2) uncomfortable |
|  | (3) slightly uncomfortable |
|  | (4) barely uncomfortable |
|  | (5) not uncomfortable |
| 5. When I am adequately active, it costs: | (1) too much money |
|  | (2) much money |
|  | (3) slightly too much money |
|  | (4) barely too much money |
|  | (5) not too much money |
| 6. When I am adequately active, it feels: | (1) very annoying |
|  | (2) annoying |
|  | (3) slightly annoying |
|  | (4) barely annoying |
|  | (5) not annoying |
| 7. When I am adequately active, it costs: | (1) a lot of effort |
|  | (2) effort |
|  | (3) slightly any effort |
|  | (4) barely any effort |
|  | (5) no effort |
| 8. When I am adequately active, I feel ashamed: | (1) very much |
|  | (2) much |
|  | (3) slightly |
|  | (4) barely |
|  | (5) not |
| 9. When I am sufficiently active, I get: | (1) very tired |
|  | (2) tired |
|  | (3) slightly tired |
|  | (4) barely tired |
|  | (5) not tired |
| 10. When I am adequately active, I feel: | (1) very observed |
|  | (2) observed |
|  | (3) slightly observed |
|  | (4) barely observed |
|  | (5) not observed |

| Social norms: item 1, 3, 5, 7  Social modeling: item 2, 4, 6, 8 | |
| --- | --- |
| 1. My partner | (1) certainly thinks that I need to be adequately active |
|  | (2) probably thinks that I need be adequately active |
|  | (3) does not have an opinion on that |
|  | (4) does probably not think that I need to be adequately active |
|  | (5) does certainly not think that I need to be adequately active |
|  | (6) I don’t have a partner |
| 2. My partner is adequately active | (1) I totally agree |
|  | (2) I agree |
|  | (3) Undecided |
|  | (4) I don’t agree |
|  | (5) Totally disagree |
|  | (6) I don’t have a partner |
| 3. Most of my family members | (1) certainly think that I need to be adequately active |
|  | (2) probably think that I need be adequately active |
|  | (3) do not have an opinion on that |
|  | (4) do probably not think that I need to be adequately active |
|  | (5) do certainly not think that I need to be adequately active |
|  | (6) I don’t have any family members/Not applicable |
| 4. Most of my family members are adequately active. | (1) I totally agree |
|  | (2) I agree |
|  | (3) Undecided |
|  | (4) I don’t agree |
|  | (5) Totally disagree |
|  | (6) I don’t have any family members/Not applicable |
|  |  |
| 5. Most of my friends | (1) certainly think that I need to be adequately active |
|  | (2) probably think that I need be adequately active |
|  | (3) do not have an opinion on that |
|  | (4) do probably not think that I need to be adequately active |
|  | (5) do certainly not think that I need to be adequately active |
|  | (6) I don’t have any friends/Not applicable |
|  |  |
| 6. Most of my friends are adequately active. | (1) I totally agree |
|  | (2) I agree |
|  | (3) Undecided |
|  | (4) I don’t agree |
|  | (5) Totally disagree |
|  | (6) I don’t have any friends/Not applicable |
| 7. Most of my colleagues | (1) certainly think that I need to be adequately active |
|  | (2) probably think that I need be adequately active |
|  | (3) do not have an opinion on that |
|  | (4) do probably not think that I need to be adequately active |
|  | (5) do certainly not think that I need to be adequately active |
|  | (6) I don’t have any colleagues/Not applicable |
| 8. Most of my colleagues are sufficiently active. | (1) I totally agree |
|  | (2) I agree |
|  | (3) Undecided |
|  | (4) I don’t agree |
|  | (5) Totally disagree |
|  | (6) I don’t have any colleagues/Not applicable |

| Self-efficacy: items 1-9 | | | | | |
| --- | --- | --- | --- | --- | --- |
| I find it hard/easy to be adequately physically active when… | | | | | |
|  | Very difficult (1) | Difficult  (2) | Neutral  (3) | Easy  (4) | Very easy  (5) |
| 1. the weather is bad |  |  |  |  |  |
| 2. I find the activity boring |  |  |  |  |  |
| 3. I have pain during the activity |  |  |  |  |  |
| 4. I have to be active on my own |  |  |  |  |  |
| 5. I am busy with many things |  |  |  |  |  |
| 6. I am tired |  |  |  |  |  |
| 7. I am stressed |  |  |  |  |  |
| 8. I feel down |  |  |  |  |  |
| 9. I dislike the activity |  |  |  |  |  |

| Intention: items 1-3 |  |
| --- | --- |
| 1. I intend to be sufficiently physically active within the next three months. | (1) Yes, absolutely |
|  | (2) Yes |
|  | (3) Neutral |
|  | (4) No |
|  | (5) No, not at all |
| 2. I am motivated to be sufficiently physically active within the next three months. | (1) Totally agree |
|  | (2) Agree |
|  | (3) Uncertain |
|  | (4) Disagree |
|  | (5) Totally disagree |
| 3. Chances that I will be sufficiently physically active within the next three months are | (1) Very little |
|  | (2) Little |
|  | (3) Medium |
|  | (4) High |
|  | (5) Very high |
